# NHERF1 Loss Upregulates Enzymes of the Pentose Phosphate Pathway in Kidney Cortex

**DOI:** 10.3390/antiox9090862

**Published:** 2020-09-14

**Authors:** Adrienne Bushau-Sprinkle, Michelle T. Barati, Kenneth B. Gagnon, Syed Jalal Khundmiri, Kathleen Kitterman, Bradford G. Hill, Amanda Sherwood, Michael Merchant, Shesh N. Rai, Sudhir Srivastava, Barbara Clark, Leah Siskind, Michael Brier, Jessica Hata, Eleanor Lederer

**Affiliations:** 1 Department of Pharmacology and Toxicology, University of Louisville, Louisville, KY 40202, USA; ambush03@louisville.edu (A.B.-S.); leah.siskind@louisville.edu (L.S.); michael.brier@louisville.edu (M.B.); 2 Department of Medicine, Division of Nephrology, University of Louisville, Louisville, KY 40202, USA; michelle.barati@louisville.edu (M.T.B.); kenneth.gagnon@louisville.edu (K.B.G.); kathleen.kitterman@louisville.edu (K.K.); amanda.sherwood@louisville.edu (A.S.); michael.merchant@louisville.edu (M.M.); 3 Department of Physiology and Biophysics, Howard University, Washington, DC 20059, USA; syed.khundmiri@Howard.edu; 4 Department of Medicine, Division of Environmental Medicine, University of Louisville, Louisville, KY 40202, USA; bradford.hill@louisville.edu; 5 Department of Bioinformatics and Biostatistics, School of Public Health Information Sciences, University of Louisville, Louisville, KY 40202, USA; shesh.rai@louisville.edu; 6 Biostatistics and Bioinformatics Facility, School of Medicine, University of Louisville, Louisville, KY 40202, USA; sudhir.srivastava@louisville.edu; 7 Centre for Agricultural Bioinformatics, ICAR-Indian Agricultural Statistics Research Institute, New Delhi 110012, India; 8 Department of Biochemistry and Molecular Genetics, University of Louisville, Louisville, KY 40202, USA; barbara.clark@louisville.edu; 9 Department of Pathology, University of Louisville, Louisville, KY 40202, USA; jessica.hata@louisville.edu; 10 Department of Pathology, Norton Children’s Hospital, Louisville, KY 40202, USA; 11 Robley Rex VA Medical Center, Louisville, KY 40206, USA

**Keywords:** cellular redox state, oxidative stress, mitochondrial function, cisplatin nephrotoxicity

## Abstract

(1) Background: We previously showed Na/H exchange regulatory factor 1 (NHERF1) loss resulted in increased susceptibility to cisplatin nephrotoxicity. NHERF1-deficient cultured proximal tubule cells and proximal tubules from NHERF1 knockout (KO) mice exhibit altered mitochondrial protein expression and poor survival. We hypothesized that NHERF1 loss results in changes in metabolic pathways and/or mitochondrial dysfunction, leading to increased sensitivity to cisplatin nephrotoxicity. (2) Methods: Two to 4-month-old male wildtype (WT) and KO mice were treated with vehicle or cisplatin (20 mg/kg dose IP). After 72 h, kidney cortex homogenates were utilized for metabolic enzyme activities. Non-treated kidneys were used to isolate mitochondria for mitochondrial respiration via the Seahorse XF24 analyzer. Non-treated kidneys were also used for LC-MS analysis to evaluate kidney ATP abundance, and electron microscopy (EM) was utilized to evaluate mitochondrial morphology and number. (3) Results: KO mouse kidneys exhibit significant increases in malic enzyme and glucose-6 phosphate dehydrogenase activity under baseline conditions but in no other gluconeogenic or glycolytic enzymes. NHERF1 loss does not decrease kidney ATP content. Mitochondrial morphology, number, and area appeared normal. Isolated mitochondria function was similar between WT and KO. Conclusions: KO kidneys experience a shift in metabolism to the pentose phosphate pathway, which may sensitize them to the oxidative stress imposed by cisplatin.

## 1. Introduction

Although cisplatin is a widely used chemotherapeutic that treats a variety of solid malignant tumors (e.g., ovarian testicular, head and neck, and lung cancer), its nephrotoxicity limits its use [1]. Twenty percent to 30% of patients on cisplatin will develop cisplatin-induced acute kidney injury (AKI) with a single dose [1]. The mechanisms underlying cisplatin’s nephrotoxicity remain perplexing. Furthermore, with no methods for the prevention or treatment of cisplatin nephrotoxicity, the identification of host factors (biomarkers) that confer susceptibility to cisplatin and new therapeutic targets for the prevention and/or treatment of cisplatin nephrotoxicity are promising areas of research.

The accumulation and bioactivation of cisplatin to a more nephrotoxic metabolite underlies the kidney’s susceptibility to cisplatin-induced AKI. Cisplatin nephrotoxicity has been found to alter renal cell metabolism and induce cell death via both apoptosis and necrosis. However, the mechanism by which cisplatin induces these pathways remains unclear. Several studies have found that cisplatin treatment leads to renal tubular cell depletion of amino acids [2,3,4,5], influences lipid metabolism through the reduction of fatty acid oxidation resulting in the accumulation of fatty acids in kidney tissue [2,5,6], and decreases glycolytic enzymes and intermediates of the pentose phosphate pathway and the citric acid cycle [2,7]. Reactive oxygen species (ROS) and mitochondrial function may also contribute to cisplatin’s mechanism of injury [8]. Mitochondria continuously produce ROS, such as superoxide, and scavenge these ROS using antioxidant enzymes (superoxide dismutase, glutathione peroxidase, catalase, and glutathione S-transferase) [9]. Cisplatin has been found to also accumulate in the mitochondria of renal epithelial cells [10,11], resulting in mitochondrial damage including decreased mitochondrial mass, disruption of cristae, and even mitochondrial swelling [12,13,14]. These morphological changes are associated with a significant reduction in mitochondrial activity and ATP production [12,13]. This structural damage to mitochondria leads to electron leakage and produces massive amounts of free radicals in the form of ROS, leading to cell injury and death.

We have recently published the novel finding that kidneys lacking the scaffolding protein Na/H exchange regulatory factor 1 (NHERF1) show increased susceptibility to cisplatin-induced AKI [15]. NHERF1 is a known monomeric membrane-associated protein that belongs to the NHERF family of post-synaptic density protein 9595/Drosophila Discs Large/ZonulaOcculens -1(PSD-95/DIg/ZO-1)homology (PDZ)-scaffold proteins [16]. NHERF1 is found in all epithelial cells and acts as a scaffold for multi-protein signaling complexes. In proximal tubule cells of the kidney, NHERF1 is anchored to the cytoskeleton in the subapical plasma membrane, where it acts as a key scaffolding protein of transport proteins and has critical roles in defining the renal proximal tubule brush border membrane (BBM) composition and in regulating ion transport [16]. Recent studies have uncovered increasing evidence that NHERF1 has a much broader role than as a scaffolding protein. In fact, alterations in NHERF1 expression, phosphorylation status, and/or localization have been associated with tumorigenesis [17,18,19], changes in cell structure and trafficking [19,20,21], inflammatory responses [19,22], and tissue injury [15,19,23,24]. These studies highlight the fact that changes in NHERF1 expression can affect cell proliferation through alterations in the WNT/beta-catenin pathway and cyclin D kinase pathways and that the absence of NHERF1 diminishes the inflammatory response by impairing the assembly of specific components of the response such as the intracellular adhesion molecule 1 (ICAM1) [23]. Previous studies from our laboratory comparing wild-type (WT) and NHERF1-deficient opossum kidney (OK) cells, a model of mammalian renal proximal tubule, demonstrated that NHERF1-deficient cells grew more slowly and were more likely to die in culture; however, they exhibited no overt morphological differences. The NHERF1-deficient cells did show decreased BBM expression of the type IIa sodium phosphate cotransporter (Npt2a), the sodium-dependent glucose cotransporter (SGLT1), and the enzyme γ-glutamyl transferase (GGTase) (unpublished data), which are defects that reversed when NHERF1 was re-introduced into the cells [25]. Likewise, NHERF1-deficient mice show normal kidney morphology but a marked decrease in the BBM expression of Npt2a, resulting in significant phosphate wasting, hypophosphatemia, hypercalciuria, and stone formation [26]. Formal studies to address metabolic changes in NHERF1-deficient kidneys have not been performed, but proteomic analysis from our laboratory comparing BBM from WT and NHERF1 KO kidneys revealed significant differences in the expression of mitochondrial proteins and enzymes from a variety of metabolic pathways. Notably, similar differences in BBM protein expression from the intestine of NHERF1 KO mice have been reported, but again, no specific studies to determine whether these changes in protein expression translate into changes in metabolism have been performed [27].

These observations suggest the hypothesis that alterations in renal cell metabolic pathways and/or mitochondrial dysfunction resulting from the loss of NHERF1 could prime these kidneys for a ‘second hit’ with cisplatin, therefore sensitizing these cells to cisplatin nephrotoxicity. The goals of this manuscript were twofold: (1) to determine if NHERF1 KO mice demonstrate changes in renal metabolic pathways, and (2) to determine if NHERF1 KO mice have altered mitochondrial function and/or structure that predisposes these animals to cisplatin nephrotoxicity.

## 2. Materials and Methods

### 2.1. Animals and Treatments

Two to 4-month-old male NHERF1^(−/−)^ KO mice [28] and their WT littermates on C57BL/6J background were maintained on a 12:12 h light–dark cycle and were provided water and food ad libitum. At the time of sacrifice, animals were anesthetized with ketamine/xylazine (100/15 mg/kg, intraperitoneally (IP)). For enzyme kinetic assays, mice were given a single IP injection of 20 mg/kg cisplatin or vehicle (saline). Vehicle-treated and cisplatin-treated mice were euthanized after 72 h. All cisplatin studies were performed at the same time each day. Kidneys were removed and decapsulated; then, the cortex was separated from the medulla for homogenization. The kidney cortex was homogenized in 0.1 M Tris-HCl, pH 7.4 on ice. Then, tissue homogenates were sonicated on ice for 10 s/sample. Then, the samples were centrifuged for 15 min at 2500× *g* at 4 °C. For the mitochondrial studies, mice were not given a cisplatin or saline injection, and kidneys were removed and decapsulated for mitochondrial isolation or snap frozen in liquid nitrogen and stored at −80 °C for ATP analysis. All animal studies were approved by the Institutional Animal Care Use Committee (IACUC) at the University of Louisville and followed the guidelines of the American Veterinary Medical Association.

### 2.2. Fructose-1,6-Bisphosphatase (FBPase) Activity Assay

FBPase activity was measured as described previously [29]. Briefly, 20 μg protein (10 μL) was added to 170 μL assay buffer containing 0.1 M Tris-HCl, pH 8.6; 0.1 MgCl_2_; 0.1 M cysteine HCl in a 96-well plate and incubated at 37 °C for 5 min. Then, 20 μL of freshly prepared 50 mM fructose-1,6-bisphosphate (final concentration 5 mM) was added. Samples along with blanks (protein and substrate) and freshly prepared standards (0, 10, 25, 50, 100, 150, 200, 250 nM KH_2_PO_4_) were incubated at 37 °C for 60 min. The reaction was stopped by the addition of 50 μL 10% trichloroacetic acid (TCA), and the samples were centrifuged at 10,000× *g* for 5 min. Then, 200 μL of supernatant from samples, standards, and blanks was carefully pipetted into a fresh 96-well plate. Then, 50 μL freshly prepared 5% FeSO_4_ (0.5 g of FeSO4 dissolved in 1 mL of 10% ammonium molybdate in 0.2 N H_2_SO_4_ and the total volume was brought to 10 mL using distilled water) was added. The samples, standards, and blanks were read after 10 min at 820 nm wavelength. All samples, blanks, and standards were read in triplicate. The amount of inorganic phosphate released by the reaction of FBPase was determined by subtracting the substrate blank values from the sample values using the standards curve. All samples, blanks, and standards were read in triplicate. The average activity from the triplicate was considered as *n* = 1 and shown as activity per mg protein (WT vehicle *n* = 3), (NHERF1 KO vehicle *n* = 4), (WT cisplatin *n* = 3), (NHERF1 KO cisplatin *n* = 5).

### 2.3. Glucose-6-Phosphatase (G6Pase) Activity Assay

G6Pase activity was measured as described [29]. Briefly, 20 μg protein (10 μL) was added to 170 μL assay buffer containing 0.1 M Tris-HCl, pH 7.4, and 0.1 M MgCl_2_ in a 96-well plate and incubated for 5 min at 37 °C. Then, 20 μL freshly prepared substrate (50 mM glucose-6-phosphate, final concentration 5 mM) was added to each well. Samples along with blanks (protein and substrate) and freshly prepared standards (0, 10, 25, 50, 100, 150, 200, 250 nM KH_2_PO_4_) were incubated at 37 °C for 60 min. The reaction was stopped by the addition of 50 μL 10% TCA, and the samples were centrifuged at 10,000× *g* for 5 min. Then, 200 μL of supernatant from samples, standards, and blanks was carefully pipetted into a fresh 96-well plate. Then, 50 μL freshly prepared 5% FeSO_4_ (0.5 g of FeSO_4_ dissolved in 1 mL of 10% ammonium molybdate in 0.2 N H_2_SO_4_ and the total volume was brought to 10 mL using distilled water) was added. The samples, standards, and blanks were read after 10 min at 820 nm wavelength. All samples, blanks, and standards were read in triplicate. The amount of inorganic phosphate released by the reaction of G6Pase was determined by subtracting the substrate blank values from the samples values using the standard curve. The average activity from triplicates was considered as *n* = 1 and shown as activity per mg protein (WT vehicle *n* = 3), (NHERF1 KO vehicle *n* = 4), (WT cisplatin *n* = 3), (NHERF1 KO cisplatin *n* = 5).

### 2.4. Lactate Dehydrogenase (LDH) Activity Assay

LDH activity was measured as described [29]. Briefly, samples (20 μg protein, 10 μL) were added to 170 μL assay buffer containing 0.1 M Tris-HCl, pH 7.4; 0.1 M MgCl_2_ and 0.5 mM sodium pyruvate (substrate) and incubated at 37 °C for 5 min. Reaction was started by adding 20 μL 5 mM nicotinamide adenine dinucleotide and hydrogen (NADH) freshly prepared in 1 mM sodium bicarbonate, pH 9.0 solution (final concentration 0.5 mM). Samples and blanks (solution, protein, and NADH) were immediately read at 340 nm for 10 min. The optical density values were recorded every minute. The optical density measured before the addition of NADH was considered as time zero. Absorbance in the linear range was used for calculating activity. The activity was determined as a decrease in absorbance due to the oxidation of NADH per mg protein. The values of NADH blank were subtracted from samples to calculate the final activity. All samples were read in triplicate and the average value was considered *n* = 1 (WT vehicle *n* = 3), (NHERF1 KO vehicle *n* = 4), (WT cisplatin *n* = 3), (NHERF1 KO cisplatin *n* = 5).

### 2.5. Malate Dehydrogenase (MDH) Activity Assay

MDH activity is determined as described [29]. Briefly, a 10 μL sample (20 μg protein was added to assay buffer containing 0.1 M Tris-HCl, pH 7.4; 0.5 mM oxaloacetate; 0.1 M MgCl_2_. Reaction was started by adding 20 μL 5 mM NADH freshly prepared in 1 mM sodium bicarbonate, pH 9.0 solution (final concentration 0.5 mM). Samples and blanks (solution, protein, and NADH) were immediately read at 340 nm for 10 min. The optical density values were recorded every minute. The optical density measured before the addition of NADH was considered as time zero. Absorbance in the linear range was used for calculating activity. The activity was determined as a decrease in absorbance due to the oxidation of NADH per mg of protein. The values of the NADH blank were subtracted from samples to calculate the final activity. All samples were read in triplicate, and the average value was considered *n* = 1 (WT vehicle *n* = 3), (NHERF1 KO vehicle *n* = 4), (WT cisplatin *n* = 3), (NHERF1 KO cisplatin *n* =5).

### 2.6. Malic Enzyme (ME) Activity Assay

ME activity was measured as described [29]. Briefly, a 10 μL sample (20 μg protein) was added to 170 μL assay buffer containing 0.1 M Tris-HCl, pH 7.4; 0.5 mM L-malic acid; 0.1 M MgCl_2_. Reaction was started by the addition of 20 μL freshly prepared 2 mg/mL of NADP^+^ in 100 mM imidazole. Samples and blanks (solution, sample, and NADP^+^ blank) were immediately read at 340 nm for 10 min. The optical density values were recorded every minute. The optical density measured before the addition of NADP^+^ was considered as time zero. Absorbance in the linear range was used for calculating activity. The activity was determined as an increase in absorbance due to the reduction of NADP^+^ to NADPH per mg protein. The values of NADP^+^ blank were subtracted from samples to calculate the final activity. All samples were read in triplicate, and the average value was considered *n* = 1 (WT vehicle *n* = 3), (NHERF1 KO vehicle *n* = 4), (WT cisplatin *n* = 3), (NHERF1 KO cisplatin *n* = 5).

### 2.7. Glucose-6-Phosphate Dehydrogenase (G6PD) Activity Assay

G6PD activity was measured as described [29]. Briefly, a 10 μL sample (20 μg protein) was added to 170 μL assay buffer containing 0.1 M Tris-HCl, pH 7.4; 0.5 mM glucose-6-phosphate; 0.1 M MgCl_2_. Reaction was started by the addition of 20 μL freshly prepared 2 mg/mL of NADP^+^ in 100 mM imidazole. Samples and blanks (solution, sample, and NADP^+^ blank) were immediately read at 340 nm for 10 min. The optical density values due to the conversion of NADP^+^ to NADPH were recorded every minute. The optical density measured before the addition of NADP^+^ was considered as time zero. Absorbance in the linear range was used for calculating activity. The activity was determined as increase in absorbance due to the reduction of NADP^+^ to NADPH per mg protein. The values of NADP^+^ blank were subtracted from samples to calculate the final activity. All samples were read in triplicate, and the average was considered *n* = 1 (WT vehicle *n* = 3), (NHERF1 KO vehicle *n* = 4), (WT cisplatin *n* = 3), (NHERF1 KO cisplatin *n* = 5).

### 2.8. Liquid Chromatography-Mass Spectrometry of Kidney Cortex for ATP Quantification

First, 10 mg of kidney cortex was snap-frozen and stored at −80 °C until the LC-MS procedure. The tissue was maintained in liquid nitrogen during the preparation for LC-MS. Tubes and beads were pre-cooled in liquid nitrogen and 2.5% kidney cortex homogenate was made using an extraction solution (70% acetonitrile (ACN) and 30% H_2_O). Tissue was homogenized using a bead beater at 5 m/s for 15 s. Then, the homogenized tissue was transferred to another tube for centrifugation at 16,000×*g* for 10 min at 4 °C. The supernatant was saved for subsequent LC-MS analysis. To quantitate the ATP present in mouse kidney cortex tissue, a standard curve was prepared using 1 mM ATP diluted serially with the extraction buffer containing 20 µM ^13^C_10_ATP. Each concentration point was diluted 50x with 60% ACN and 40% 15 mM ammonium acetate. Then, 5 µL was injected onto a SeQuant ZIC-cHILIC 100X2.1 mm metal-free HPLC column. Separation was performed by pumping 60% ACN and 40% 15 mM ammonium acetate through the column with a flow rate of 0.5 mL/min with no change in composition using a Waters Acquity UPLC. ATP was eluted from the column and flowed into a Waters Quattro Premier XE mass spectrometer and subsequently quantitated using optimized MRMs. A calibration curve was constructed using ATP concentration on the x-axis and the response of ATP over ^13^C_10_ATP on the y-axis. Mouse kidney extracts were also diluted with 50x 60% ACN and 40% 15 mM ammonium acetate, 5 µL was injected, and the concentration in kidney tissue was interpolated using the calibration curve previously made for (WT *n* = 5) and (NHERF1 KO *n* = 5).

### 2.9. Perfusion Fixation of Total Kidney In Situ for Electron Microscopy

The abdominal cavity of the mouse was opened along the *linea alba* and intestines were moved aside to expose kidneys. A suture was loosely put around the right kidney for removal without glutaraldehyde exposure. Then, the right kidney was used for kidney cortex homogenates. Next, the chest cavity was opened to expose the heart for perfusion through the left ventricle, and the vena cava was cut, while the right kidney was tied off and removed. The left mouse kidney was perfused with 3% glutaraldehyde solution at a rate of 6 mL/min for approximately 1–3 min. Perfusion was stopped once kidneys had a change in color and consistency. The kidney was removed and placed in a petri dish of 3% glutaraldehyde where three to four 0.2 cm × 0.4 cm slices were cut and then stored in 3–4 mL of 3% glutaraldehyde at 4 °C. At this point, the tissue was sent to Norton Children’s Hospital Pathology Department for a blind EM analysis of kidney tubule mitochondria. Images were taken by a renal pathologist at the Pathology Department. Sections with the highest concentration of mitochondria were randomly picked in a 4x field to be used to calculate mitochondria number and mitochondria area by Image J (WT *n* = 6) and (NHERF1 KO *n* = 5).

### 2.10. Seahorse XF24 Mitochondrial Respiration Analysis

Mice for this study had food taken away 6 h before sacrifice. Mitochondrial oxidative capacity was measured in isolated kidney mitochondria using a Seahorse Bioscience XF24 extracellular flux analyzer (Billerica, MA, USA). For measurements in isolated mitochondria, tissue from the kidney cortex of both kidneys (approximately 50 mg) was isolated and homogenized in 1 mL of isolation buffer (220 mM mannitol, 70 mM sucrose, 5 mM 3-(N-morpholino) propanesulfonic acid (MOPS), 1 mM ethylene glycol tetraacetic acid (EGTA), 0.3% fatty acid-free bovine serum albumin (BSA), pH 7.2). The homogenate was centrifuged at 500× *g* for five minutes at 4 °C. The supernatant containing mitochondria was centrifuged at 10,000× *g* for five minutes. Following two wash centrifugation steps in BSA-free isolation buffer, the mitochondria were suspended in respiration buffer (120 mM KCl, 25 mM sucrose, 10 mM 4-(2-hydroxyethyl)-1-piperazineethanesulfonic acid (HEPES), 1 mM MgCl_2_, 5 mM KH_2_PO_4_, pH to 7.2). Protein in the mitochondrial suspension was estimated by the bicinchoninic acid method (Sigma) using BSA as a standard. Then, 10 μg of mitochondrial protein was sedimented in XF culture plates. Succinate (10 mM), rotenone (1 μM), and ADP (1 mM) were injected to assess state 3 respiratory activity (phosphorylating respiration). The oxygen consumption rate (OCR) of mitochondria after exposure to oligomycin (1 μg/mL) was used to estimate state 4 activity (non-phosphorylating respiration). Finally, Antimycin A (20 μM) was utilized to stop all respiration. Data are expressed as pmol O_2_/min/μg protein (WT *n* = 6, NHERF1 KO *n* = 6).

### 2.11. Brush Border Membrane Isolation

Kidney cortex BBM from WT and NHERF1 KO mice were prepared at 4 °C using the MgCl_2_ precipitation method as previously described [30]. Kidney cortex slices were minced and then briefly homogenized in 50 mM mannitol and 5 mM Tris-HEPES buffer, pH 7.0 (20 mL/g), in a glass teflon homogenizer with four complete strokes. Then, a polyron homogenizer was used to provide high-speed [20,500 revolutions/min (rpm)] homogenization for three strokes of 15 s each with an interval of 15 s between each stroke. MgCl_2_ was added to the homogenate to a final concentration of 10 mM and slowly stirred for 20 min. The homogenate was spun at 2000× *g* in a Sorvall high-speed centrifuge using an SS-34 rotor. The supernatant was centrifuged at 35,000× *g* for 30 min using the SS-34 rotor. Then, the pellet was resuspended in 300 mM mannitol and 5 mM Tris-HEPES, pH 7.4, with four passes by a loose-fitting Dounce homogenizer (Wheaton, IL) and centrifuged at 35,000× *g* for 20 min in 15 mL Corex tubes using the SS-34 rotor. The outer final pellet was resuspended in a small volume of buffered 300 mM mannitol.

### 2.12. Label-Free Quantitative Liquid Chromatography-Mass Spectrometry

Protein samples were analyzed by 2D-LC-MS/MS using either a linear trap quadrupole (LTQ) ion trap mass spectrometer (Thermo Fisher Scientific, Waltham, MA, USA) and/or using a Thermo Scientific LTQ Orbitrap Elite hybrid FTMS system enabled with electron transfer dissociation (ETD) fragmentation. Routinely one-dimensional (1D) reversed phase (RP) or two-dimensional (2D) strong cation exchange (SCX)-(RP) HPLC experiments were conducted off line for the LC-MALDI approach or online for the LC-LTQ-XL-Orbitrap-ESI-MS/MS approach and protein assignments were made as previously described [31,32,33,34].

The acquired mass spectrometry data were searched against an appropriate REFSEQ protein database using the SEQUEST (version 27 revision 11) algorithm and Matrix Science Mascot v.1.4 using Proteome Discoverer v1.3 to prepare and process RAW files as well as aggregate results. Separately for comparative LCMS analyses, the MS/MS database analysis was performed with SequestSorcerer (Sage-N Research, San Jose, CA) and high-probability peptide and protein identifications were assigned from the SEQUEST results using the Protein and PeptideProphet (tools.proteomecenter.org/software.php) and SageN Sorcerer statistical platforms. Scaffold 3 proteomic analysis software (ProteomeSoftware, Inc, Portland, OR) was used for quantitative comparison using a label-free spectral counting method. The qualitative comparison of protein expression patterns was performed by Ingenuity Pathways Analysis software (http://ingenuity.com).

### 2.13. Interpretation of Protein Assignment Results

Lists of identified proteins and expression patterns were submitted for pathways analysis using one of several publically available (David, http://david.abcc.ncifcrf.gov/; GO, http://www.geneontology.org/; KEGG Pathway database, http://genome.jp/kegg/pathway.html), GenMAPP (www.genmapp.org/) or commercially licensed tools (Ingenuity Pathways Analysis software, (Redwood City, California, USA) http://ingenuity.com).

### 2.14. Statistical Analysis

Data are shown as means ± standard error means (SEM) or standard deviation (SD). Two-way ANOVA was used to compare the different treatment groups for the enzyme kinetic assays. Student’s t-test was performed for the LC-MS data measuring ATP content, mitochondrial number, mitochondrial area, and mitochondrial respiration via Seahorse XF24. All statistical analysis was conducted with SPSS version 24 software. *p* values of < 0.05 were considered statistically significant.

## 3. Results

### 3.1. Absence of NHERF1 Results in Extensive Changes in Kidney BBM Protein Expression

BBM were prepared from the kidney cortex of WT and NHERF1 KO mice, followed by comparative proteomic analysis. Pathway analysis of differentially expressed proteins revealed significant changes in proteins associated with mitochondrial function as well as protein components of signaling pathways, actin cytoskeleton, cell survival, and oxidative phosphorylation (Supplementary Materials Figure S1, Table S1). Based on these findings, we proceeded to determine if metabolic pathways of WT and NHERF1 KO mouse kidneys differed. Under normal conditions, gluconeogenesis is a signature metabolic pathway for kidney proximal tubules with little glycolysis [35]. Therefore, we examined enzymes of the pathways involved in carbohydrate metabolism.

### 3.2. Cisplatin Treatment Significantly Decreases FBPase and G6Pase Enzyme Activity in Both WT and NHERF1 KO Mice

**Figure 1:**
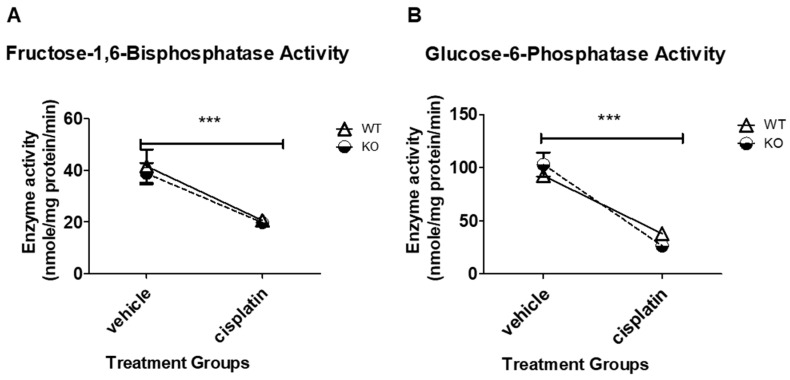
Effect of cisplatin treatment on fructose-1,6-bisphosphatase and glucose-6-phosphase enzyme activity in wild-type (WT) and Na/H exchange regulatory factor 1 (NHERF1) knockout (KO) mouse kidneys. Two to 4-month-old male C57BL/6J WT and NHERF1 KO mice were given cisplatin (20 mg/kg dose intraperitoneally (IP)) or vehicle (saline) and sacrificed after 72 h as described in the Methods section. (**A**) Fructose-1,6-bisphosphatase (FBPase) enzyme activity was determined from the kidney cortex tissue of these mice. Data are means ± SEM (WT vehicle *n* = 3), (KO vehicle *n* = 4), (WT cisplatin *n* = 3), and (KO cisplatin *n* = 5). *** *p* = 0.001 cisplatin-treated WT and NHERF1 KO mice compared to vehicle saline controls. (**B**) Glucose-6-phosphatase (G6Pase) enzyme activity was determined from the kidney cortex tissue of these mice. Data are means ± SEM (WT vehicle *n* = 3), (KO vehicle *n* = 4), (WT cisplatin *n* = 3), and (KO cisplatin *n* = 5). *** *p* < 0.001 cisplatin-treated WT and NHERF1 KO mice compared to vehicle saline controls.

Previous studies investigated the effect of cisplatin on gluconeogenesis [2,36,37]; however, the effect of NHERF1 protein loss on gluconeogenesis has not been investigated. FBPase is a critical regulatory enzyme in gluconeogenesis that catalyzes the hydrolysis of fructose-1,6-bisphosphate to fructose-6-phosphate and inorganic phosphate [38]. In order to determine if NHERF1 loss affected gluconeogenic enzyme activity alone or with cisplatin treatment, male 2–4-month-old mice were treated with vehicle or cisplatin to induce AKI and then sacrificed after 72 h. Kidney cortex tissue from these mice were used for the FBPase enzyme kinetic assay as described in the methods section. There were no significant differences between vehicle [(WT: 41.5 nmole/mg protein/min ± 6.5) (KO: 38.7 nmole/mg protein/min ± 4.1)] or cisplatin [(WT: 20.6 nmole/mg protein/min ± 0.4) (KO: 19.7 nmole/mg protein/min ± 1.2)] treated WT and NHERF KO kidneys (Figure 1A). However, cisplatin did decrease FBPase activity in both WT and NHERF1 KO kidneys (*p* = 0.0001) (Figure 1A).

G6Pase is the final step in gluconeogenesis, where it hydrolyzes glucose-6-phosphate to free glucose and a phosphate group [39]. Similarly to FBPase, G6Pase activity was comparable in WT and NHERF1 KO kidneys regardless of treatment [(WT vehicle: 92.3 nmole/mg protein/min ± 5.0), (KO vehicle: 103.0 nmole/mg protein/min ± 11.3), (WT cisplatin: 38.0 nmole/mg protein/min ± 5.1), and (KO cisplatin: 26.4 nmole/mg protein/min ± 3.8)]. Cisplatin led to a significant decrease in enzyme activity in both genotypes (*p* < 0.0001) (Figure 1B).

### 3.3. NHERF1 Deficiency or Cisplatin Treatment Does Not Significantly Affect LDH or MDH Enzyme Activity in Mice

**Figure 2:**
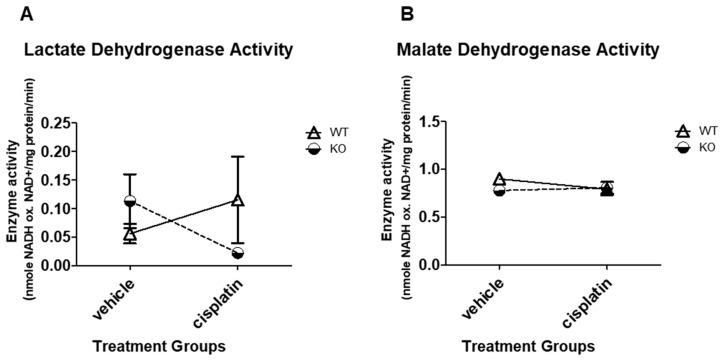
Lactate dehydrogenase and malate dehydrogenase enzyme activity in WT and NHERF1 KO mouse kidneys. Two to 4-month-old male C57BL/6J WT and NHERF1 KO mice were given cisplatin (20 mg/kg dose IP) or vehicle (saline) and sacrificed after 72 h as described in the Methods section. (**A**) Lactate dehydrogenase (LDH) enzyme activity was determined from kidney cortex tissue of these mice. Data are mean ± SEM (WT vehicle *n* = 3), (KO vehicle *n* = 4), (WT cisplatin *n* = 3), and (NHERF1 KO cisplatin *n* = 5). No significant differences were recorded. (**B**) Malate dehydrogenase (MDH) enzyme activity was determined from the kidney cortex tissue of these mice. Data are mean ± SEM (WT vehicle *n* = 3), (KO vehicle *n* = 4), (WT cisplatin *n* = 3), (NHERF1 KO cisplatin *n* = 5). No significant differences were reported.

Lactate dehydrogenase (LDH) catalyzes the interconversion of lactate and pyruvate, concomitantly with the interconversion of NADH and NAD^+^ [40]. When oxygen is absent, LDH converts pyruvate, the final product of glycolysis, to lactate [40]. Thus, LDH activity was measured in vehicle and cisplatin mouse kidneys. NHERF1 loss (*p* = 0.65) or cisplatin treatment (*p* = 0.71) did not significantly affect lactate dehydrogenase activity in these mouse kidneys [(WT vehicle: 0.06 nmole/mg protein/min ± 0.02), (KO vehicle: 0.1 nmole/mg protein/min ± 0.05), (WT cisplatin: 0.1 nmole/mg protein/min ± 0.8), and (KO cisplatin: 0.02 nmole/mg protein/min ± 0.006)] (Figure 2A).

Malate dehydrogenase (MDH) is an enzyme involved in many metabolic pathways including the citric acid cycle. MDH reversibly catalyzes the oxidation of malate to oxaloacetate with the reduction of NAD^+^ to NADH [41]. In this study, we measured MDH activity as the conversion of oxaloacetate to malate and oxidation of NADH to NAD^+^. The effect of NHERF1 loss and/or cisplatin treatment on MDH activity was analyzed. In a similar way to LDH, NHERF1 loss or cisplatin treatment did not significantly affect MDH activity in these mouse kidneys [(WT vehicle: 0.9 nmole/mg protein/min ± 0.06), (KO vehicle: 0.7 nmole/mg protein/min ± 0.02), (WT cisplatin: 0.8 nmole/mg protein/min ± 0.02), and (KO cisplatin: 0.81 nmole/mg protein/min ± 0.06)] (Figure 2B).

### 3.4. NHERF1 Deficiency Upregulates ME and G6PD Activity

**Figure 3:**
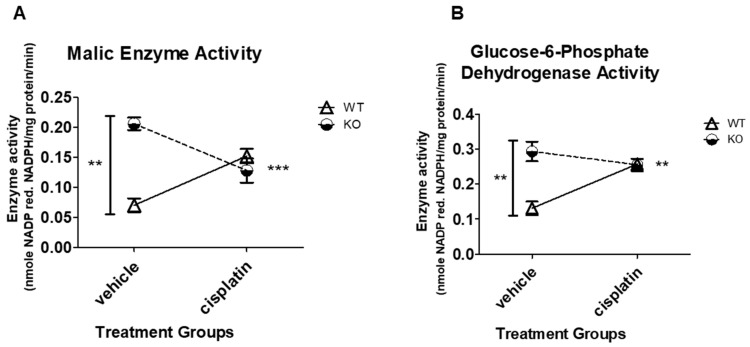
Effect of NHERF1 loss and cisplatin treatment on malic enzyme and glucose-6-phosphate dehydrogenase enzyme activity in WT and NHERF1 KO mouse kidneys. Two to 4-month-old male C57BL/6J WT and NHERF1 KO mice were given cisplatin (20 mg/kg dose IP) or vehicle (saline) and sacrificed after 72 h as described in the Methods section. (**A**) Malic enzyme (ME) activity was determined from the kidney cortex tissue of these mice. Data are mean ± SEM (WT vehicle *n* = 3), (KO vehicle *n* = 4), (WT cisplatin *n* = 3), and (KO cisplatin *n* = 5). ** *p* = 0.0065. Vehicle-treated NHERF1 KO mice compared to WT vehicle controls; *** *p* = 0.0005 interaction of cisplatin-treated NHERF1 KO mice to cisplatin-treated WT mice. (**B**) G6PD enzyme activity was determined from the kidney cortex tissue of these mice. Data are mean ± SEM (WT vehicle *n* = 3), (KO vehicle *n* = 4), (WT cisplatin *n* = 3), and (KO cisplatin *n* = 5). ** *p* = 0.0033 vehicle-treated NHERF1 KO mice compared to WT vehicle controls; ** *p* = 0.00029 interaction of cisplatin-treated NHERF1 KO mice to cisplatin-treated WT mice.

Malic enzyme (ME) catalyzes the conversion of malic acid to pyruvate and produces NADPH [42]. ME serves as an additional source of NADPH for lipogenesis. In order to understand the effect that NHERF1 loss and/or cisplatin treatment may have on ME activity, kidney cortex tissue from vehicle or cisplatin-treated WT and NHERF1 KO were evaluated. Interestingly, there was a significant genotype effect on ME activity resulting in an increase in activity in NHERF1 KO kidneys (*P* = 0.0065) (Figure 3A). Additionally, a significant interaction was also noted between WT and NHERF1 KO kidneys after cisplatin treatment (*p* = 0.0005) [(WT vehicle: 0.07 nmole/mg protein/min ± 0.012), (KO vehicle: 0.21 nmole/mg protein/min ± 0.01), (WT cisplatin: 0.15 nmole/mg protein/min ± 0.012), and (KO cisplatin: 0.13 nmole/mg protein/min ± 0.02)] (Figure 3A).

Cisplatin-induced AKI is known to decrease intermediates of the pentose phosphate pathway [2,7] in mice. Glucose-6-phosphate dehydrogenase (G6PD) is a cytosolic enzyme that participates in the pentose phosphate pathway, resulting in NADPH production [43]. This is accomplished when G6PD reduces NADP^+^ to NADPH while oxidizing glucose-6-phosphate [43]. G6PD enzyme activity was analyzed in vehicle and cisplatin-treated WT and NHERF1 KO kidney cortex to elucidate if NHERF1 loss and/or cisplatin treatment affected the pentose phosphate pathway. Similarly, to ME, there was a significant genotype effect on G6PD activity, resulting in an increase in activity in NHERF1 KO kidneys (*p* = 0.0033) (Figure 3B). Additionally, a significant interaction was also noted between WT and NHERF1 KO kidneys after cisplatin treatment (*p* = 0.00029) [(WT vehicle: 0.13 nmole/mg protein/min ± 0.02), (KO vehicle: 0.3 nmole/mg protein/min ± 0.03), (WT cisplatin: 0.3 nmole/mg protein/min ± 0.007), and (KO cisplatin: 0.3 nmole/mg protein/min ± 0.02)] (Figure 3B).

### 3.5. NHERF1 Deficiency Does Not Affect ATP Abundance in Mouse Kidneys

**Figure 4:**
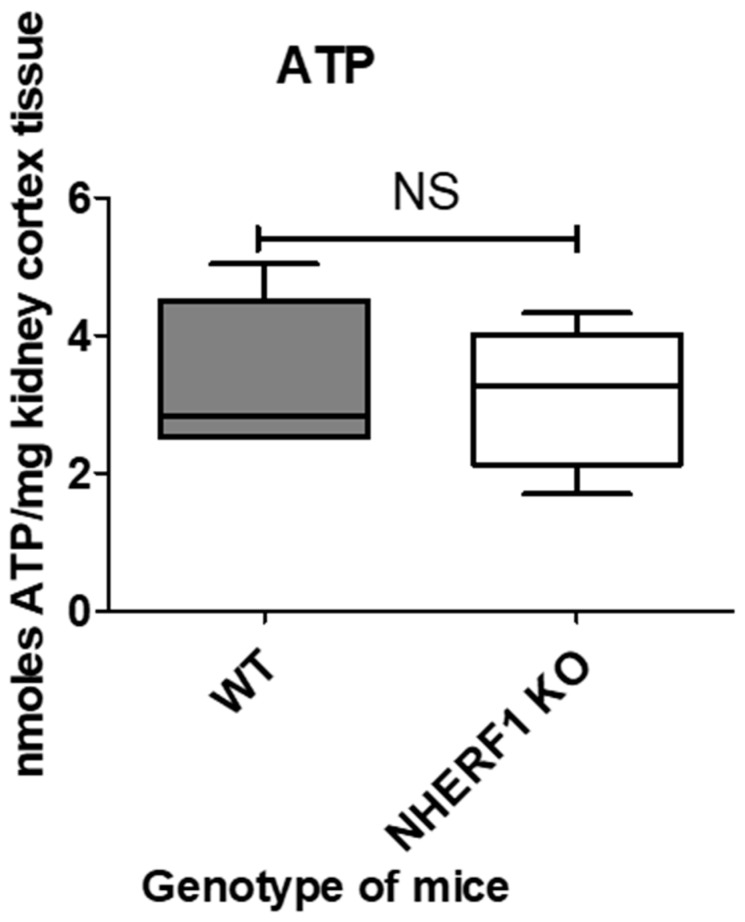
ATP content of WT and NHERF1 KO mouse kidneys.LC-MS was utilized to evaluate the amount of ATP in these tissues as described in the Methods section. Data are means ± SEM (WT *n* = 5) and (KO *n* = 5). No significant differences were reported in these kidneys.

ATP provides energy to drive many cellular processes and is consumed during many metabolic processes [44]. In eukaryotes, ATP is produced by three different metabolic pathways: [1] glycolysis, [2] the citric acid cycle or oxidative phosphorylation, and [3] beta-oxidation [44]. In order to determine if NHERF1 KO kidneys had differences in ATP content, kidneys were snap-frozen and processed while cold for LC-MS as described in the Methods section. LC-MS analysis revealed there were no significant differences in ATP amount in WT (3.4 nmoles/mg tissue ± 0.5) and NHERF1 KO (3.1 nmoles/mg tissue ± 0.5) kidneys (*p* = 0.67) (Figure 4).

### 3.6. NHERF1 Deficiency Does Not Affect Kidney Proximal Tubule Mitochondria Morphology, Number, or Area

The mitochondrial structure is essential for proper function; thus, EM images of WT and NHERF1 KO proximal tubule mitochondria were utilized to evaluate their morphology. These images were of 2–4-month-old male C57BL/6J WT and NHERF1 KO mice whose kidneys were perfused with 3% glutaraldehyde prior to EM analysis.

**Figure 5:**
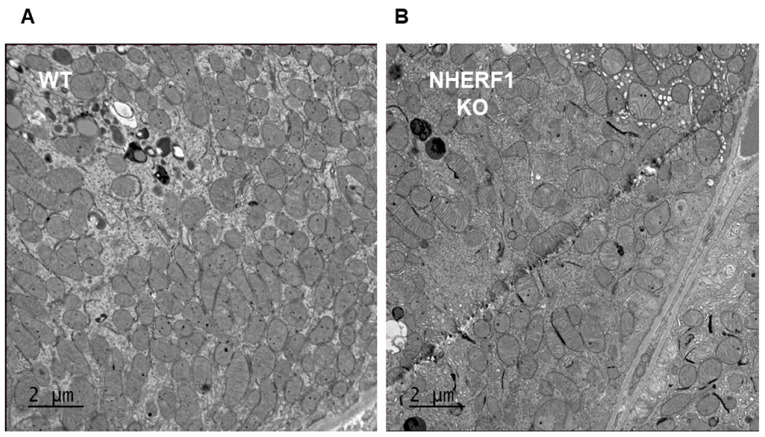
Electron microscopy of mitochondria in WT and NHERF1 KO proximal tubules.

These were taken and evaluated by a renal pathologist for signs of injury, oxidative stress, and changes in cristae. There were no apparent changes in mitochondria morphology between WT and NHERF1 KO proximal tubules (Figure 5, panels A and B). The only injury reported was early ischemic changes most likely due to harvesting of the kidneys (Figure 5). Some endosomal swelling was noted but occurred across both genotypes. Additionally, the density and distribution of mitochondria within the tubules were alike, and no apparent signs of oxidative stress were found in either genotype (Figure 5).

**Figure 6:**
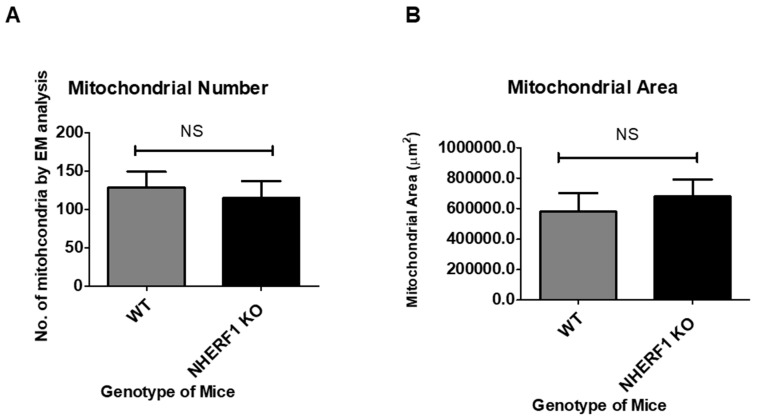
Evaluation of mitochondrial number and area of WT and NHERF1 KO proximal tubules. (**A**) Number of mitochondria were counted in random 4× visual fields with the highest density of mitochondria. Data are means ± SEM (WT *n* = 6) and (NHERF1 KO *n* = 5). The mitochondria number of NHERF1 KO proximal tubules was insignificant when compared to WT. (**B**) Mitochondria area was calculated using electron microscopy (EM) images and Image J. Data are means ± SEM (WT *n* = 6) and (NHERF1 KO *n* = 5). Mitochondria area of NHERF1 KO proximal tubules were insignificant when compared to WT.

Changes in mitochondrial number and a decrease in size have been associated with a decline in mitochondrial function [45]. Therefore, one goal of this study was to determine if mitochondrial number and/or size changed in NHERF1 KO proximal tubules when compared to WT. Images from electron microscopy (EM) of WT and NHERF1 KO kidney proximal tubules were utilized in order to count the number of mitochondria and to calculate the average area via Image J. There was not a significant difference between the average number of mitochondria between WT and NHERF1 KO tubules (WT average number: 128.8) and (NHERF1 KO average number: 115) (*p* = 0.6) (Figure 6A). In addition, there was not a significant difference between the average area of mitochondria in WT and NHERF1 KO tubules (WT average area: 580,540.9 μm^2^) and (NHERF1 KO average area: 678,465.4 μm^2^) (*p* = 0.75) (Figure 6B).

### 3.7. WT and NHERF1 KO Mouse Kidney Mitochondria Have Similar Oxidative Capacities

**Figure 7:**
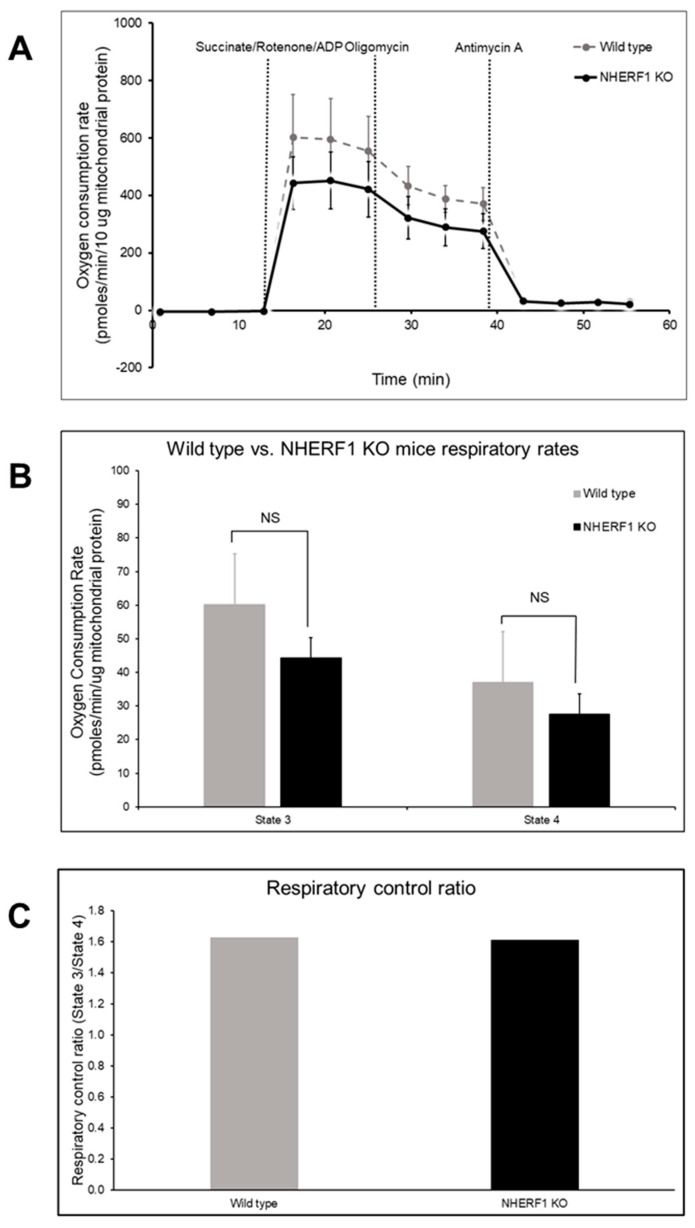
Mitochondrial function in isolated mitochondria of WT and NHERF1 KO kidneys. Mitochondria from two to 4-month-old male WT and NHERF1 KO mice were isolated and analyzed via the Seahorse XF24 for oxidative capacity as described in the Methods section. (**A**) Oxygen consumption rate (OCR) was recorded after the addition of both substrates (succinate/rotenone/ADP) and inhibitors (oligomycin and antimycin A) [(WT *n* = 6) and (NHERF1 KO *n* = 6)]. (**B**) State 3 and state 4 were calculated using the recorded OCRs of WT and NHERF1 KO mitochondria. Data are mean ± SD [(WT *n* = 6) and (NHERF1 KO *n* = 6)]. State 3 and state 4 respiration were considered insignificant between WT and NHERF1 KO mouse kidney mitochondria. (**C**) Respiratory control ratio (RCR) (state 3/state 4) was calculated between WT and NHERF1 KO kidney mitochondria. Data are represented as state 3/state 4 ratio [(WT *n* = 6) and (NHERF1 *n* = 6)]. RCR was insignificant between WT and NHERF1 KO mouse kidney mitochondria.

The mitochondria’s capacity to reduce oxygen is a critical aspect in the process of mitochondrial electron transport and ATP synthesis. Therefore, measuring mitochondrial oxygen consumption can provide a valuable method to assess mitochondrial function. One purpose of this work was to assess mitochondrial function by oxidative capacity in WT and NHERF1 KO kidneys using the Seahorse XF24 analyzer. In panel A of Figure 7, the oxygen consumption rate (OCR) of WT and NHERF1 KO kidney mitochondria are shown over time. Both WT and NHERF1 KO mitochondria exhibit a similar trend and response to added substrates and inhibitors. When adding the substrate Succinate/Rotenone plus ADP for the production of ATP, both genotypes exhibit a maximal increase in OCR. Moreover, both genotypes undergo a decrease in OCR after adding oligomycin, an inhibitor of complex V (formation of ATP from ADP via O_2_ consumption). Lastly, antimycin A shuts down all respiration, where the OCR is close to the basal OCR. The difference between the basal OCR and OCR after antimycin A is the non-mitochondrial respiration.

Changes in state 3 (conversion of ATP from ADP and consumption of O_2_) and state 4 (non-phosphorylating or resting respiration) respiration are commonly used to evaluate mitochondria oxidative capacity. Panel B of Figure 7 shows both state 3 [(WT: 60 pmoles/min/μg protein ± 15) and (NHERF1 KO: 44 pmoles/min/μg protein ± 6)] and state 4 [(WT: 37 pmoles/min/μg protein ± 15) and (NHERF1 KO: 28 pmoles/min/μg protein ± 6)] of WT and NHERF1 KO kidney mitochondria, where state 3 (*p* = 0.2) and 4 (*p* = 0.1) was determined to not be significantly different between the groups.

The respiratory control ratio (RCR) is the best general measure of mitochondrial function in isolated mitochondria. RCR is measured by taking state 3/state 4 respiration and sums up the main function of mitochondria: the ability to respond to ADP from a resting state by making ATP at high rates. The RCR has no absolute value that is diagnostic of mitochondrial dysfunction [46]. Thus, RCR values are substrate and tissue-dependent, making the RCR advantageous when measuring mitochondrial function in isolated mitochondria [46]. A change in almost any aspect of oxidative phosphorylation will result in a change in the RCR when comparing isolated mitochondria [46].

Representative photomicrographs show a 4x field of WT and NHERF1 KO proximal tubule mitochondria. Panel A represents the proximal tubule mitochondria of WT mice, while panel B represents NHERF1 KO proximal tubule mitochondria (WT *n* = 6) and (NHERF1 KO *n* = 5). The scale bars were set at 2 μm.

Accordingly, the RCR was calculated between WT (1.63) and NHERF1 (1.61) KO kidney mitochondria and was also found to not be significantly different (Figure 7C).

## 4. Discussion

This work aimed to examine two aspects of proximal tubule cell function: metabolic enzymatic pathways and mitochondrial structure and function. We proposed that changes in kidney metabolism and/or mitochondrial structure or function could predispose NHERF1 KO mice to cisplatin nephrotoxicity. This is the first study to find changes in the kidney pentose phosphate pathway enzymes with NHERF1 loss and a novel proposed mechanism of susceptibility to cisplatin-induced AKI. Recent studies have found that cisplatin alters renal cell metabolism, contributing to injury and the secondary result of chronic kidney disease (CKD) development [2,3,4,5,6]. Cisplatin treatment results in the depletion of amino acids in the kidney [2,3,4,5], reduces fatty acid oxidation while concomitantly accumulating fatty acids in the kidney [2,5,6], and decreases renal glycolytic enzymes and intermediates of the pentose phosphate pathway [2,36]. In addition to affecting metabolic pathways cisplatin, nephrotoxicity has been established in inducing apoptotic and necrotic cell death. The mechanisms involved in cisplatin-induced nephrotoxic cell death remain unclear. However, there is increasing evidence that ROS and mitochondrial function have an important role in cisplatin’s mechanism of injury. These observations combined with the increased susceptibility to cisplatin-induced AKI suggested the hypothesis that NHERF1 KO mice have metabolic alterations and/or mitochondrial dysfunction that predispose them to cisplatin nephrotoxicity.

NHERF1 KO mice did not exhibit changes in gluconeogenic or glycolytic enzyme activity. Indeed, cisplatin treatment resulted in a parallel decrease in FBPase and G6Pase activity in NHERF1 KO and WT mice. Additionally, there were no significant changes with LDH and MDH activity between non-treated and treated WT and NHERF1 KO mice. These results are in agreement with previous studies [2,7]. Interestingly, NHERF1 KO mouse kidneys exhibit increased activity of ME and G6PD under baseline conditions when compared to WT mouse kidneys. The significance of this shift in metabolism toward a greater utilization of the pentose phosphate pathway is not entirely clear. However, these findings suggest a potential compensatory mechanism for increased NADPH production as protection against oxidative stress. ME and G6PD activity provide necessary NADPH, a key cofactor in redox control and reductive biosynthesis. ME plays a role in the production of pyruvate and serves as an additional source of NADPH for lipogenesis. Additionally, there is recent evidence for direct cross-talk between ME and the pentose phosphate pathway [47], where G6PD is a rate-limiting enzyme. A study using a cell culture model of diabetes observed that the increased activity of G6PD restored redox balance in endothelial cells exposed to high glucose levels [48], where high glucose levels had previously decreased G6PD and increased levels of oxidative stress. A similar observation has been made in studies of liver cirrhosis in rats subject to oxidative stress, where an increase in ME and G6PD gene expression and activity [49] are also seen, presumably providing protection against the stress through an increased production of NADPH [49]. Multiple studies have noted the importance of cellular redox balance in both the development of and in protection from renal injury [50,51]. Additionally, one other investigator found that NHERF1 is a previously unidentified regulator of Nox1 (NADPH oxidase) and promotes Nox1 activity [52]. Taken together, these observations suggest that the kidneys of NHERF1 KO mice experience a greater degree of oxidative stress that is masked by increased NADPH production through the pentose phosphate pathway. If NHERF1 KO mouse kidneys are more reliant on the pentose phosphate pathway for maintenance of the cellular redox state, the decrease in the activity of the enzymes of the pentose phosphate pathway resulting from cisplatin toxicity could potentially result in more severe injury.

While proteomic data demonstrated changes in mitochondrial proteins in NHERF1 KO mice (Figure S1) (Table S1), we found no alterations in mitochondrial structure or function. The kidney ATP levels in NHERF1 KO mice are equivalent to WT. EM analysis revealed that NHERF1 KO mice have similar proximal tubule mitochondrial morphologies, size, distribution and number when compared to WT. This result is not surprising, as our proteomic analysis did not identify differences in any of the proteins associated with mitochondrial fission or fusion such as dynamin related protein 1 (Drp1), mitofusin-1 (Mfn1), or mitofusin-2 (Mfn2). Mitochondria from NHERF1 KO and WT kidneys were found to have similar oxidative capacities as demonstrated by the measurement of OCR and RCR (WT: 1.63 and KO: 1.61) (Figure 7C). However, these findings do not exclude completely a role for altered mitochondrial function as a contributor to enhanced susceptibility to cisplatin-induced AKI. Although isolated mitochondria from NHERF1 KO kidneys function normally, they may not do so in intact tissue. NHERF1 KO mice undergo phosphate wasting [28] due to the faulty trafficking of Npt2a to the apical membrane [21], which may result in intracellular phosphate deficiency. The loss of intracellular phosphate may create an intracellular environment where mitochondria cannot function properly. NHERF1 KO mouse kidneys may also sustain losses of other important nutrients. In addition to higher urine phosphate excretion, NHERF1 KO mice also demonstrate hypercalciuria and hyperuricosuria [26]. A full evaluation of the alterations in proximal tubule cell transport in the NHERF1 KO kidneys has not been examined. The absence of NHERF1 may also result in impaired signaling processes [53], alterations in intracellular or mitochondrial protein phosphorylation, loss of mitochondrial interaction with other organelles, or aberrant mitochondrial protein localization, as suggested by the proteomic data demonstrating changes in mitochondrial proteins associated with the renal cortical BBM (Figure S1) (Table S1). Thus, further studies to compare mitochondrial function of WT and NHERF1 KO in intact kidney tissue are warranted.

## 5. Conclusions

In conclusion, this study provides insight into metabolic and mitochondrial changes of NHERF1 KO mice and provides new avenues to explore regarding NHERF1 loss and susceptibility to cisplatin nephrotoxicity. We did not find changes in enzyme activity in gluconeogenesis or the citric acid cycle, ATP content, and mitochondrial morphology and function. However, we discovered that enzymes of the pentose phosphate pathway were found to be increased in NHERF1 KO mice and suggest these animals are expressing some differences in metabolic pathways and may be compensating for an underlying stress. The basis for these changes in the activity of this metabolic pathway and its significance for the increased susceptibility of NHERF1 KO mice to cisplatin nephrotoxicity remain unknown. These results provide another area to be explored in the future pertaining to NADPH levels in NHERF1 KO mouse kidneys. Further investigation into the bioenergetics of NHERF1 KO mouse kidneys may elucidate more insight into susceptibility to cisplatin injury and increase our understanding of the underlying mechanism of susceptibility to cisplatin-induced AKI. In the future, this information may provide novel therapeutic targets and/or biomarkers to use clinically for the prevention of cisplatin nephrotoxicity.

## Supplementary Materials

The following are available online at https://www.mdpi.com/2076-3921/9/9/862/s1, Figure S1: Proteomic analysis of BBM from WT and NHERF1 KO mice, Table S1: Differential expression of BBM proteins from WT and NHERF1 KO mice.

## Data Sharing

Data files for acquired LCMS data (.RAW) and scaffold search results (.sf3) files along with a sample key and sequence database were deposited in MassIVE (http://massive.ucsd.edu/) data repository (MassIVE ID: MSV000085816) with the Center for Computational Mass Spectrometry at the University of California, San Diego and shared with the ProteomeXchange (www.proteomexchange.org) (Proteome Exchange ID: PXD020534).

## References

[1] Hanigan M.H., Devarajan P. (2003). Cisplatin nephrotoxicity: Molecular mechanisms. Cancer Ther..

[2] Lagies S., Pichler R., Kaminski M.M., Schlimpert M., Walz G., Lienkamp S.S., Kammerer B. (2018). Metabolic characterization of directly reprogrammed renal tubular epithelial cells (iRECs). Sci. Rep..

[3] Zhang P., Chen J.-Q., Huang W.-Q., Li W., Huang Y., Zhang Z.-J., Xu F.-G. (2017). Renal Medulla is More Sensitive to Cisplatin than Cortex Revealed by Untargeted Mass Spectrometry-Based Metabolomics in Rats. Sci. Rep..

[4] Boudonck K.J., Mitchell M.W., Német L., Keresztes L., Nyska A., Shinar D., Rosenstock M. (2009). Discovery of Metabolomics Biomarkers for Early Detection of Nephrotoxicity. Toxicol. Pathol..

[5] Portilla D., Li S., Nagothu K.K., Megyesi J., Kaissling B., Schnackenberg L., Safirstein R.L., Beger R.D. (2006). Metabolomic study of cisplatin-induced nephrotoxicity. Kidney Int..

[6] Wilmes A., Bielow C., Ranninger C., Bellwon P., Aschauer L., Limonciel A., Chassaigne H., Kristl T., Aiche S., Huber C.G. (2015). Mechanism of cisplatin proximal tubule toxicity revealed by integrating transcriptomics, proteomics, metabolomics and biokinetics. Toxicology In Vitro.

[7] Choi Y.M., Kim H.K., Shim W., Anwar M.A., Kwon J.W., Kwon H.K., Kim H.J., Jeong H., Kim H.M., Hwang D. (2015). Mechanism of Cisplatin-Induced Cytotoxicity Is Correlated to Impaired Metabolism Due to Mitochondrial ROS Generation. PLoS ONE.

[8] Ueda N., Kaushal G.P., Shah S.V. (2000). Apoptotic mechanisms in acute renal failure. Am. J. Med..

[9] Richter C., Gogvadze V., Laffranchi R., Schlapbach R., Schweizer M., Suter M., Walter P., Yaffee M. (1995). Oxidants in mitochondria: From physiology to diseases. Biochim. Et Biophys. Acta.

[10] Singh G. (1989). A possible cellular mechanism of cisplatin-induced nephrotoxicity. Toxicology.

[11] Gemba M., Fukuishi N. (1991). Amelioration by ascorbic acid of cisplatin-induced injury in cultured renal epithelial cells. Contrib. Nephrol..

[12] Oh G.-S., Kim H.-J., Shen A., Lee S.-B., Yang S.-H., Shim H., Cho E.-Y., Kwon K.-B., Kwak T.H., So H.-S. (2016). New Therapeutic Concept of NAD Redox Balance for Cisplatin Nephrotoxicity. Biomed. Res. Int..

[13] Yang Y., Liu H., Liu F., Dong Z. (2014). Mitochondrial dysregulation and protection in cisplatin nephrotoxicity. Arch. Toxicol..

[14] Zsengellér Z.K., Ellezian L., Brown D., Horváth B., Mukhopadhyay P., Kalyanaraman B., Parikh S.M., Karumanchi S.A., Stillman I.E., Pacher P. (2012). Cisplatin nephrotoxicity involves mitochondrial injury with impaired tubular mitochondrial enzyme activity. J. Histochem. Cytochem..

[15] Bushau-Sprinkle A., Barati M., Conklin C., Dupre T., Gagnon K.B., Khundmiri S.J., Clark B., Siskind L., Doll M.A., Rane M. (2019). Loss of the Na(+)/H(+) Exchange Regulatory Factor 1 Increases Susceptibility to Cisplatin-Induced Acute Kidney Injury. Am. J. Pathol..

[16] Khundmiri S.J., Weinman E.J., Steplock D., Cole J., Ahmad A., Baumann P.D., Barati M., Rane M.J., Lederer E. (2005). Parathyroid hormone regulation of NA+,K+-ATPase requires the PDZ 1 domain of sodium hydrogen exchanger regulatory factor-1 in opossum kidney cells. J. Am. Soc. Nephrol. Jasn.

[17] Dai J.L., Wang L., Sahin A.A., Broemeling L.D., Schutte M., Pan Y. (2004). NHERF (Na+/H+ exchanger regulatory factor) gene mutations in human breast cancer. Oncogene.

[18] Georgescu M.M., Morales F.C., Molina J.R., Hayashi Y. (2008). Roles of NHERF1/EBP50 in cancer. Curr. Mol. Med..

[19] Bushau-Sprinkle A.M., Lederer E.D. (2020). New roles of the Na(+)/H(+) exchange regulatory factor 1 scaffolding protein: A review. Am. J. Physiology. Ren. Physiol..

[20] Sun L., Zheng J., Wang Q., Song R., Liu H., Meng R., Tao T., Si Y., Jiang W., He J. (2016). NHERF1 regulates actin cytoskeleton organization through modulation of alpha-actinin-4 stability. FASEB J. Off. Publ. Fed. Am. Soc. Exp. Biol..

[21] Ketchem C.J., Khundmiri S.J., Gaweda A.E., Murray R., Clark B.J., Weinman E.J., Lederer E.D. (2015). Role of Na+/H+ exchanger regulatory factor 1 in forward trafficking of the type IIa Na+-Pi cotransporter. Am. J. Physiology. Ren. Physiol..

[22] Jiang Y., Lu G., Trescott L.R., Hou Y., Guan X., Wang S., Stamenkovich A., Brunzelle J., Sirinupong N., Li C. (2013). New conformational state of NHERF1-CXCR2 signaling complex captured by crystal lattice trapping. PLoS ONE.

[23] Li M., Mennone A., Soroka C.J., Hagey L.R., Ouyang X., Weinman E.J., Boyer J.L. (2015). Na(+) /H(+) exchanger regulatory factor 1 knockout mice have an attenuated hepatic inflammatory response and are protected from cholestatic liver injury. Hepatology.

[24] Leslie K.L., Song G.J., Barrick S., Wehbi V.L., Vilardaga J.P., Bauer P.M., Bisello A. (2013). Ezrin-radixin-moesin-binding phosphoprotein 50 (EBP50) and nuclear factor-kappaB (NF-kappaB): A feed-forward loop for systemic and vascular inflammation. J. Biol. Chem..

[25] Khundmiri S.J., Ahmad A., Bennett R.E., Weinman E.J., Steplock D., Cole J., Baumann P.D., Lewis J., Singh S., Clark B.J. (2008). Novel regulatory function for NHERF-1 in Npt2a transcription. Am. J. Physiology. Ren. Physiol..

[26] Weinman E.J., Mohanlal V., Stoycheff N., Wang F., Steplock D., Shenolikar S., Cunningham R. (2006). Longitudinal study of urinary excretion of phosphate, calcium, and uric acid in mutant NHERF-1 null mice. Am. J. Physiology. Ren. Physiol..

[27] Donowitz M., Singh S., Singh P., Salahuddin F.F., Chen Y., Chakraborty M., Murtazina R., Gucek M., Cole R.N., Zachos N.C. (2010). Alterations in the proteome of the NHERF1 knockout mouse jejunal brush border membrane vesicles. Physiol. Genom..

[28] Shenolikar S., Voltz J.W., Minkoff C.M., Wade J.B., Weinman E.J. (2002). Targeted disruption of the mouse NHERF-1 gene promotes internalization of proximal tubule sodium-phosphate cotransporter type IIa and renal phosphate wasting. Proc. Natl. Acad. Sci. USA.

[29] Khundmiri S.J., Asghar M., Khan F., Salim S., Yusufi A.N. (2004). Effect of ischemia and reperfusion on enzymes of carbohydrate metabolism in rat kidney. J. Nephrol..

[30] Khundmiri S.J., Rane M.J., Lederer E.D. (2003). Parathyroid hormone regulation of type II sodium-phosphate cotransporters is dependent on an A kinase anchoring protein. J. Biol. Chem..

[31] Merchant M.L., Powell D.W., Wilkey D.W., Cummins T.D., Deegens J.K., Rood I.M., McAfee K.J., Fleischer C., Klein E., Klein J.B. (2010). Microfiltration isolation of human urinary exosomes for characterization by MS. Proteom. Clin. Appl..

[32] Luerman G.C., Powell D.W., Uriarte S.M., Cummins T.D., Merchant M.L., Ward R.A., McLeish K.R. (2011). Identification of phosphoproteins associated with human neutrophil granules following chemotactic peptide stimulation. Mol Cell Proteom..

[33] Merchant M.L., Cummins T.D., Wilkey D.W., Salyer S.A., Powell D.W., Klein J.B., Lederer E.D. (2008). Proteomic analysis of renal calculi indicates an important role for inflammatory processes in calcium stone formation. Am. J. Physiol. Ren. Physiol..

[34] Uriarte S.M., Powell D.W., Luerman G.C., Merchant M.L., Cummins T.D., Jog N.R., Ward R.A., McLeish K.R. (2008). Comparison of proteins expressed on secretory vesicle membranes and plasma membranes of human neutrophils. J. Immunol..

[35] Mandel L.J. (1985). Metabolic substrates, cellular energy production, and the regulation of proximal tubular transport. Annu. Rev. Physiol..

[36] Zhou R., Vander Heiden M.G., Rudin C.M. (2002). Genotoxic Exposure Is Associated with Alterations in Glucose Uptake and Metabolism. Cancer Res..

[37] Hannemann J., Baumann K. (1988). Cisplatin-induced lipid peroxidation and decrease of gluconeogenesis in rat kidney cortex: Different effects of antioxidants and radical scavengers. Toxicology.

[38] Timson D.J. (2019). Fructose 1,6-bisphosphatase: Getting the message across. Biosci. Rep..

[39] Van Schaftingen E., Gerin I. (2002). The glucose-6-phosphatase system. Biochem. J..

[40] Valvona C.J., Fillmore H.L., Nunn P.B., Pilkington G.J. (2016). The Regulation and Function of Lactate Dehydrogenase A: Therapeutic Potential in Brain Tumor. Brain Pathol..

[41] Gietl C. (1992). Malate dehydrogenase isoenzymes: Cellular locations and role in the flow of metabolites between the cytoplasm and cell organelles. Biochim. Et Biophys. Acta (BBA) Bioenerg..

[42] Frenkel R., Horecker B.L., Stadtman E.R. (1975). Regulation and Physiological Functions of Malic Enzymes. Current Topics in Cellular Regulation.

[43] Efferth T., Schwarzl S.M., Smith J., Osieka R. (2006). Role of glucose-6-phosphate dehydrogenase for oxidative stress and apoptosis. Cell Death Differ..

[44] Bonora M., Patergnani S., Rimessi A., De Marchi E., Suski J.M., Bononi A., Giorgi C., Marchi S., Missiroli S., Poletti F. (2012). ATP synthesis and storage. Purinergic Signal..

[45] Chistiakov D.A., Sobenin I.A., Revin V.V., Orekhov A.N., Bobryshev Y.V. (2014). Mitochondrial aging and age-related dysfunction of mitochondria. Biomed. Res. Int..

[46] Brand M.D., Nicholls D.G. (2011). Assessing mitochondrial dysfunction in cells. Biochem. J..

[47] Yao P., Sun H., Xu C., Chen T., Zou B., Jiang P., Du W. (2017). Evidence for a direct cross-talk between malic enzyme and the pentose phosphate pathway via structural interactions. J. Biol. Chem..

[48] Zhang Z., Yang Z., Zhu B., Hu J., Liew C.W., Zhang Y., Leopold J.A., Handy D.E., Loscalzo J., Stanton R.C. (2012). Increasing glucose 6-phosphate dehydrogenase activity restores redox balance in vascular endothelial cells exposed to high glucose. PLoS ONE.

[49] Sanz N., Díez-Fernández C., Valverde A.M., Lorenzo M., Benito M., Cascales M. (1997). Malic enzyme and glucose 6-phosphate dehydrogenase gene expression increases in rat liver cirrhogenesis. Br. J. Cancer.

[50] Zunino F., Pratesi G., Micheloni A., Cavalletti E., Sala F., Tofanetti O. (1989). Protective effect of reduced glutathione against cisplatin-induced renal and systemic toxicity and its influence on the therapeutic activity of the antitumor drug. Chem. Biol. Interact..

[51] Shiraishi F., Curtis L.M., Truong L., Poss K., Visner G.A., Madsen K., Nick H.S., Agarwal A. (2000). Heme oxygenase-1 gene ablation or expression modulates cisplatin-induced renal tubular apoptosis. Am. J. Physiol. Ren. Physiol..

[52] Al Ghouleh I., Meijles D.N., Mutchler S., Zhang Q., Sahoo S., Gorelova A., Henrich Amaral J., Rodriguez A.I., Mamonova T., Song G.J. (2016). Binding of EBP50 to Nox organizing subunit p47phox is pivotal to cellular reactive species generation and altered vascular phenotype. Proc. Natl. Acad. Sci. USA.

[53] Pera T., Tompkins E., Katz M., Wang B., Deshpande D.A., Weinman E.J., Penn R.B. (2019). Specificity of NHERF1 regulation of GPCR signaling and function in human airway smooth muscle. FASEB J. Off. Publ. Fed. Am. Soc. Exp. Biol..

