# Supplementary figures and images for: NHERF1 Loss Upregulates Enzymes of the Pentose Phosphate Pathway in Kidney Cortex
Source: Antioxidants (Basel). 2020 Sep 14;9(9):862. doi: 10.3390/antiox9090862 (PMC7554817; doi:10.3390/antiox9090862)

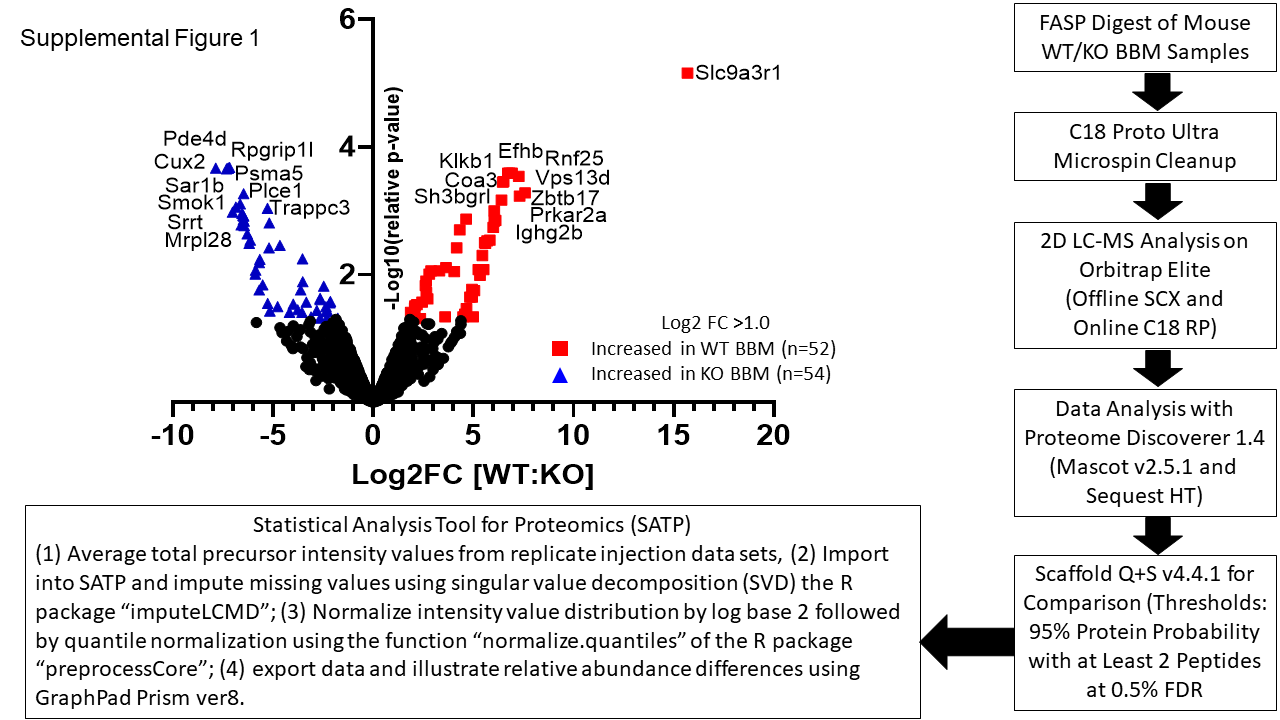

Supplement: Supplementary file 1 [file antioxidants-09-00862-s001.zip › Figure S1.TIF]
